# Generation of KCL036 research grade human embryonic stem cell line carrying a mutation in the *HTT* gene

**DOI:** 10.1016/j.scr.2016.02.026

**Published:** 2016-03

**Authors:** Laureen Jacquet, Heema Hewitson, Victoria Wood, Neli Kadeva, Glenda Cornwell, Stefano Codognotto, Carl Hobbs, Emma Stephenson, Dusko Ilic

**Affiliations:** a Stem Cell Laboratories, Division of Women's Health, Faculty of Life Sciences and Medicine, King's College London and Assisted Conception Unit, Guys' Hospital, London, United Kingdom; b Histology Laboratory, Wolfson Centre for Age-Related Diseases, Faculty of Life Sciences and Medicine, King's College London, London, United Kingdom

## Abstract

The KCL036 human embryonic stem cell line was derived from an embryo donated for research that carried an autosomal dominant mutation affecting one allele of the *HTT* gene encoding huntingtin (38 trinucleotide repeats; 14 for the normal allele). The ICM was isolated using laser microsurgery and plated on γ-irradiated human foreskin fibroblasts. Both the derivation and cell line propagation were performed in an animal product-free environment. Pluripotent state and differentiation potential were confirmed by in vitro and in vivo assays.

## Resource table

**Table:** 

Name of stem cell line	KCL036
Institution	King's College London, London UK
Derivation team	Neli Kadeva, Victoria Wood, Glenda Cornwell, Stefano Codognotto, Emma Stephenson
Contact person and email	Dusko Ilic, email: dusko.ilic@kcl.ac.uk
Date archived/stock date	Nov 22, 2011
Type of resource	Biological reagent: cell line
Sub-type	Human pluripotent stem cell line
Origin	Human embryo
Key marker expression	Pluripotent stem cell markers: NANOG, OCT4, TRA-1-60, TRA-1-81, alkaline phosphatase (AP) activity
Authentication	Identity and purity of line confirmed
Link to related literature (direct URL links and full references)	1) Ilic, D., Stephenson, E., Wood, V., Jacquet, L., Stevenson, D., Petrova, A., Kadeva, N., Codognotto, S., Patel, H., Semple, M., Cornwell, G., Ogilvie, C., Braude, P., 2012. Derivation and feeder-free propagation of human embryonic stem cells under xeno-free conditions. Cytotherapy. 14 (1), 122–128. doi: 10.3109/14,653,249.2011.623692 http://www.ncbi.nlm.nih.gov/pubmed/22029654 2) Stephenson, E., Jacquet, L., Miere, C., Wood, V., Kadeva, N., Cornwell, G., Codognotto, S., Dajani, Y., Braude, P., Ilic, D., 2012. Derivation and propagation of human embryonic stem cell lines from frozen embryos in an animal product-free environment. Nat. Protoc. 7 (7), 1366–1381. doi: 10.1038/nprot.2012.080 http://www.ncbi.nlm.nih.gov/pubmed/22722371 3) Jacquet, L., Neueder, A., Földes, G., Karagiannis, P., Hobbs, C., Jolinon, N., Mioulane, M., Sakai, T., Harding, S.E., Ilic, D., 2015. Three Huntington's Disease Specific Mutation-Carrying Human Embryonic Stem Cell Lines Have Stable Number of CAG Repeats upon In Vitro Differentiation into Cardiomyocytes. PLoS One. 10(5), e0126860. http://www.ncbi.nlm.nih.gov/pubmed/25993131
Information in public databases	KCL036 is a National Institutes of Health (NIH) registered hESC line NIH Registration Number: 0241 NIH Approval Number: NIHhESC-13-0241 http://grants.nih.gov/stem_cells/registry/current.htm?id=668
Ethics	The hESC line KCL036 is derived under license from the UK Human Fertilisation and Embryology Authority (research license numbers: R0075 and R0133) and also has local ethical approval (UK National Health Service Research Ethics Committee Reference: 06/Q0702/90). Informed consent was obtained from all subjects and the experiments conformed to the principles set out in the WMA Declaration of Helsinki and the NIH Belmont Report. No financial inducements are offered for donation.

## Resource details

**Table:** 

Consent signed	Jul 21, 2011
Embryo thawed	Oct 23, 2011
UK Stem Cell Bank Deposit Approval	Sep 13, 2012 Reference: SCSC12-28
Sex	Male 46, XY
Grade	Research
Disease status (Fig. 1)	Mutation affecting one allele of the *HTT* gene encoding huntingtin (~ 38 CAG repeats; 14 for the normal allele) associated with Huntington's disease (Ilic et al., 2015)
Karyotype (aCGH)	Decreased copy number at 2q37.3 (242,930,599–242,948,040) and at 3q25.1 (151,368,847–151,542,568). The imbalances are considered to be benign copy number variants. The chromosome 3 imbalance was not called by software.
DNA fingerprint	Allele sizes (in bp) of 17 microsatellite markers specific for chromosomes 13, 18 and 21 (Jacquet et al., 2015)
Viability testing	Pass
Pluripotent markers (immunostaining) (Fig. 2)	NANOG, OCT4, TRA-1-60, TRA-1-81, AP activity (Jacquet et al., 2015)
Three germ layers differentiation in vitro (immunostaining) (Fig. 3)	Endoderm: AFP (α-fetoprotein); Ectoderm: TUBB3 (tubulin, β3 class III); Mesoderm: ACTA2 (actin, α2, smooth muscle) (Jacquet et al., 2015)
Three germ layer differentiation in vivo (teratomas) (Fig. 4)	Endoderm: AFP, GATA4. Ectoderm: TUBB3, GFAP (glial fibrillary acidic protein). Mesoderm: DES (desmin), Alcian Blue and periodic acid–Schiff (PAS)-stained cartilage (Jacquet et al., 2015)
Targeted differentiation (Fig. 5)	Cardiomyocytes: TNNT2 (cardiac troponin T) immunostaining
Sibling lines available	None

**Fig. 2:**
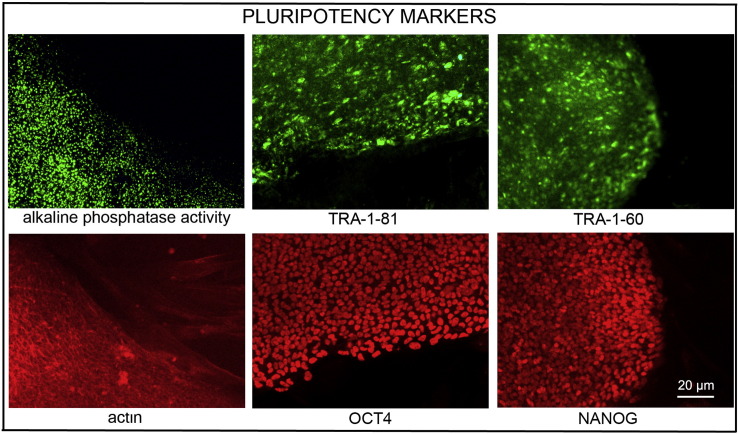
Expression of pluripotency markers. Pluripotency is confirmed by immunostaining (Oct4, Nanog, TRA-1-60, TRA-1-81) and alkaline phosphatase (AP) activity assay. Scale bar, 20 μm.

**Fig. 3:**
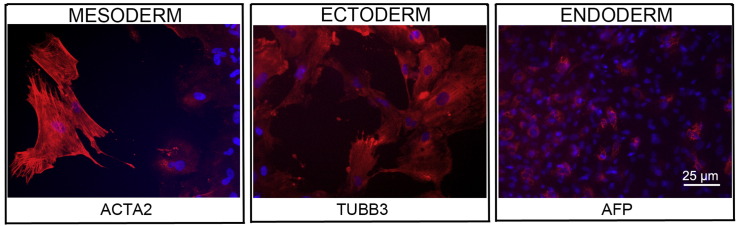
Differentiation of three germ layers in vitro is confirmed by detection of markers: smooth muscle actin (ACTA2, red) for mesoderm, β-III tubulin (TUBB3, red) for ectoderm, and α-fetoprotein (AFP, red) for endoderm. Nuclei are visualized with Hoechst 33342 (blue). Scale bar, 25 μm.

**Fig. 4:**
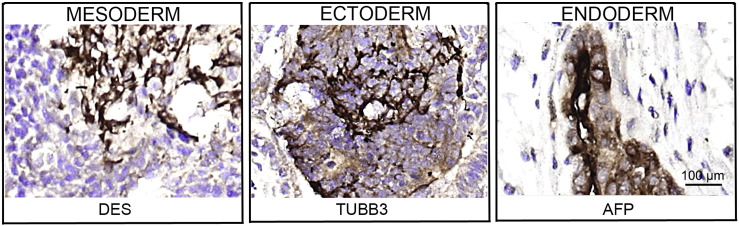
Differentiation of three germ layers in vivo. Teratomas were encapsulated and did not invade surrounding tissue. Sections are counterstained with hematoxylin and eosin and specific stains are brown (immunohistochemistry). Germ layer marker: DES for mesoderm, TUBB3 for ectoderm, and AFP for endoderm. Scale bars are 100 μm.

**Fig. 5:**
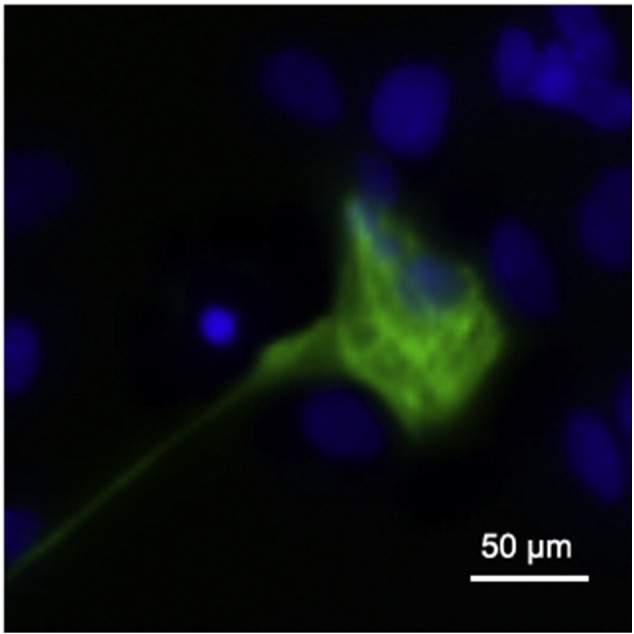
TNNT2 (green) immunostaining on day 30 of cardiac differentiation. Nuclei are visualized with Hoechst 33342 (blue). Scale bar, 50 μm.

We generated KCL036 research grade hESC line following protocols established previously (Ilic et al., 2012, Stephenson et al., 2012, Jacquet et al., 2013). The expression of the pluripotency markers was tested after freeze/thaw cycle (Fig. 2; Jacquet et al., 2015). Differentiation potential into three germ layers was verified in vitro (Fig. 3 and Fig. 5; Jacquet et al., 2015) and in vivo (Fig. 4; Jacquet et al., 2015).

Validation for sterility and specific and non-specific human pathogens confirmed that the cells in Master Bank were sterile, mycoplasma-free, and negative for human immunodeficiency virus 1 (HIV-1), Human T-lymphotropic virus type 1 (HTLV-1), hepatitis B and C (HBV and HCV), human herpes simplex virus HHV-4 (Epstein–Barr virus, EBV), and human cytomegalovirus (hCMV).

We also generated research grade of KCL036 line that is adapted to feeder-free conditions (Jacquet et al., 2015).

## Materials and methods

### Consenting process

We distribute patient information sheets (PIS) and consent forms to the in vitro fertilization (IVF) patients if they opted to donate to research embryos that were stored for 5 or 10 years. They mail signed consent back to us and that might be months after the PIS and consent were mailed to them. If in the meantime new versions of PIS/consent are implemented, we do not send these to the patients or ask them to re-sign; the whole process is done with the version that was given them initially. The PIS/consent documents (PGD-V.9) were created on Feb. 09, 2011. HFEA Code of Practice that was in effect at the time of document creation: Edition 8—R.2 (http://www.hfea.gov.uk/2999.html). The donor couple signed the consent on Jul. 21, 2011. HFEA Code of Practice that was in effect at the time of donor signature: Edition 8—R.3. HFEA Code of Practice Edition 8—R.2 was in effect: Apr. 07, 2010–Apr. 06, 2011. HFEA Code of Practice Edition 8—R.3 was in effect: Apr. 07, 2011–Oct. 01, 2011.

### Embryo culture and micromanipulation

Embryo culture and laser-assisted dissection of inner cell mass (ICM) were carried out as previously described in details (Ilic et al., 2012, Stephenson et al., 2012). The cellular area containing the ICM was then washed and transferred to plates containing mitotically inactivated human neonatal foreskin fibroblasts (HFF).

### Cell culture

ICM plated on mitotically inactivated HFF were cultured as described (Ilic et al., 2012, Stephenson et al., 2012). TE cells were removed mechanically from outgrowth (Ilic et al., 2007, Ilic et al., 2010). hESC colonies were expanded and cryopreserved at the third passage.

### Viability test

Straws with the earliest frozen passage (p.2–3) are thawed and new colonies are counted 3 days later. These colonies are then expanded up to passage 8, at which point cells were part frozen and part subjected to standard battery of tests (pluripotency markers, in vitro and in vivo differentiation capability, genetics, sterility, mycoplasma).

### Pluripotency markers

Pluripotency was assessed using two different techniques: enzymatic activity assay [alkaline phosphatase (AP) assay] and immunostaining as described (Ilic et al., 2012, Stephenson et al., 2012).

### Differentiation

Spontaneous differentiation into three germ layers was assessed in vitro and in vivo (Jacquet et al., 2015) as described (Ilic et al., 2012, Stephenson et al., 2012, Petrova et al., 2014). Targeted differentiation in cardiomyocytes followed the protocols described earlier (Jacquet et al., 2015, Laflamme et al., 2007).

### Genotyping

DNA was extracted from hESC cultures using a Chemagen DNA extraction robot according to the manufacturer's instructions. Amplification of polymorphic microsatellite markers was carried out as described (Ilic et al., 2012). Allele sizes were recorded to give a unique fingerprint of each cell line.

### Array comparative genomic hybridization (aCGH)

aCGH was performed as described in details (Ilic et al., 2012).

### Special pathology

The Doctors Laboratory London (UK) tested the line for HIV1, HepB, HepC, CMV, and EBV by PCR.

## Author disclosure statement

There are no competing financial interests in this study.

## Figures and Tables

**Fig. 1:**
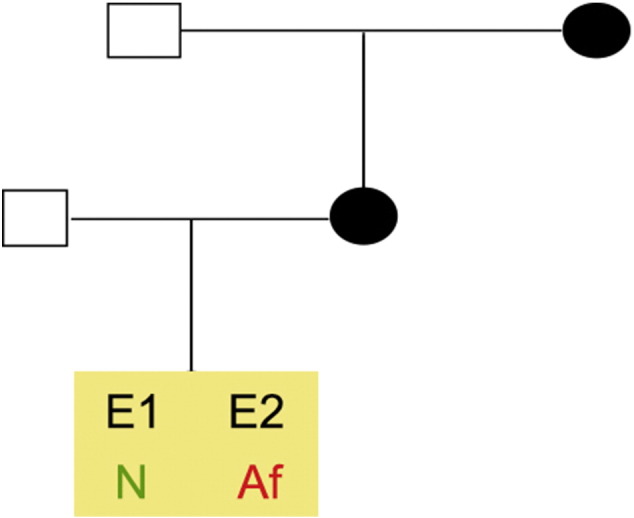
Genetic pedigree tree. The couple undergoing IVF had 16 embryos in this particular cycle. Three embryos were normal, whereas those that carried the mutation in *HTT* were donated for research. We derived hESC lines from two of them.

## References

[bb0005] Ilic D., Stephenson E., Wood V., Jacquet L., Stevenson D., Petrova A., Kadeva N., Codognotto S., Patel H., Semple M., Cornwell G., Ogilvie C., Braude P. (2012). Derivation and feeder-free propagation of human embryonic stem cells under xeno-free conditions. Cytotherapy.

[bb0010] Ilic D., Caceres E., Lu S., Julian P., Foulk R., Krtolica A. (2010). Effect of karyotype on successful human embryonic stem cell derivation. Stem Cells Dev..

[bb0015] Ilic D., Genbacev O., Krtolica A. (2007). Derivation of hESC from intact blastocysts. Curr. Protoc. Stem. Cell Biol..

[bb0020] Jacquet L., Neueder A., Földes G., Karagiannis P., Hobbs C., Jolinon N., Mioulane M., Sakai T., Harding S.E., Ilic D. (2015). Three Huntington's disease specific mutation-carrying human embryonic stem cell lines have stable number of CAG repeats upon in vitro differentiation into cardiomyocytes. PLoS ONE.

[bb0025] Jacquet L., Stephenson E., Collins R., Patel H., Trussler J., Al-Bedaery R., Renwick P., Ogilvie C., Vaughan R., Ilic D. (2013). Strategy for the creation of clinical grade hESC line banks that HLA-match a target population. EMBO Mol. Med..

[bb0030] Laflamme M.A., Chen K.Y., Naumova A.V., Muskheli V., Fugate J.A., Dupras S.K., Reinecke H., Xu C., Hassanipour M., Police S., O'sullivan C., Collins L., Chen Y., Minami E., Gill E.A., Ueno S., Yuan C., Gold J., Murry C.E. (2007). Cardiomyocytes derived from human embryonic stem cells in pro-survival factors enhance function of infarcted rat hearts. Nat. Biotechnol..

[bb0035] Petrova A., Celli A., Jacquet L., Dafou D., Crumrine D., Hupe M., Arno M., Hobbs C., Cvoro A., Karagiannis P., Devito L., Sun R., Adame L.C., Vaughan R., McGrath J.A., Mauro T.M., Ilic D. (2014). 3D in vitro model of a functional epidermal permeability barrier from human embryonic stem cells and induced pluripotent stem cells. Stem Cell Rep..

[bb0040] Stephenson E., Jacquet L., Miere C., Wood V., Kadeva N., Cornwell G., Codognotto S., Dajani Y., Braude P., Ilic D. (2012). Derivation and propagation of human embryonic stem cell lines from frozen embryos in an animal product-free environment. Nat. Protoc..

